# Adhesive Plaster

**Published:** 1875-06

**Authors:** 


ADHESIVE PLASTER.—One of the best adhesive plasters we have ever used for sores, wounds, and bruises, is made by melting together over a slow fire, and thoroughly stirring, one pound of common yellow resin; three ounces each of Burgundy pitch, yellow beeswax, and mutton tallow. Spread thinly on fine muslin, it makes excellent "adhesive strip," and does not lose its tenacity by keeping, nor crumble away from the cloth as does the kind bought of the druggist.

